# The Challenge of Teaching Essential Immunology Laboratory Skills to Undergraduates in One Month—Experience of an Osteoimmunology Course on TLR Activation

**DOI:** 10.3389/fimmu.2019.01822

**Published:** 2019-07-31

**Authors:** Teun J. de Vries, Ton Schoenmaker, Henk A. van Veen, Jolanda Hogervorst, Przemek M. Krawczyk, Carolyn G. J. Moonen, Ineke D. C. Jansen

**Affiliations:** ^1^Department of Periodontology, Academic Centre for Dentistry Amsterdam, University of Amsterdam and VU University, Amsterdam, Netherlands; ^2^Amsterdam University College, University of Amsterdam and VU University, Amsterdam, Netherlands; ^3^Department of Medical Biology, Amsterdam University Medical Centers, Location AMC, University of Amsterdam, Amsterdam, Netherlands; ^4^Department of Oral Cell Biology, Academic Centre for Dentistry Amsterdam, University of Amsterdam and VU University, Amsterdam, Netherlands

**Keywords:** laboratory work, education–active learning, osteoimmunology, toll-like receptors, osteoclast, mineralization assay

## Abstract

Acquiring immunology laboratory skills during undergraduate studies is often a prerequisite for admission to Masters’ programs. Many broad liberal arts and sciences honors degree colleges struggle in teaching these essentials since only limited time is usually reserved for this. Here, we describe a new 1-month-course developed to train a small group of honors students in 6 techniques that are useful for immunology research. In essence, 15 students were divided into 3 groups of 5 students where each student became involved in current osteoimmunology research. Osteoimmunology is a relatively new branch of the immunology tree, where the effects of inflammation and the immune system on bone formation and bone degradation is studied. A broad, 3 weeks experiment on the chronic effects of molecules that specifically activate toll-like receptors TLR2 and TLR4 on bone formation or osteoclast differentiation was performed just before the start of the course. Control samples and samples treated with TLR2 (group A), TLR4 (group B), or TLR2+TLR4 (group C) agonists were harvested and analyzed using quantitative PCR, ELISA, biochemistry, microscopy of enzyme-histochemically stained osteoclasts, scanning electron microscopy, and confocal microscopy. Each technique was taught for 2 days by a specialized instructor, who was present at all laboratory activities. The primary research question for each group was: how does the experimental condition affect bone formation or osteoclast formation? The secondary research question specified per technique was: how does this technique answer part of the primary research question? Pedagogically, students were encouraged to collaborate within the group to analyze the obtained data. Secondly, at the end of the course, a representative of each group collaborated to summarize the TLR activation modalities of a technique of choice. Thirdly, each group wrote a report, where introduction and discussion were graded as a group; each technique part was graded individually. The summary of the results from the 3 treatment modalities was presented orally. The student evaluation of the course was high, students remarked that the course had a curriculum overarching function, since it created an awareness and appreciation for both the joy and the blood-sweat-and-tears aspects of pipetting, and writing research articles, making interpretation of those easier.

## Introduction

After gaining the essential biomedical knowledge in immunology and molecular cell biology theoretical courses during the first years of an undergraduate program, there is a great urge for hands-on experience; an urge to acquire laboratory skills. This should be satisfied within the curriculum because of two reasons. (1) We should not only train theoreticians since undergraduate courses must connect properly to existing Masters' programs that demand essential laboratory skills. (2) As a teacher-scientist community, we have the obligation to convey our enthusiasm for scientific research to the next generation of biomedical researchers, providing state-of-the-art research within a laboratory context.

Can we design flexible courses, allowing the yearly incorporation of novel immunology/ molecular cell biology research insights? Can we prepare courses in such a way that undergraduate students contribute to the progression of science with visible results? Can we get students hands-on acquainted with a variety of techniques within a short time frame? We asked ourselves these challenging questions when designing the course described below. This course meets the need for flexibility in the rapidly evolving field of osteoimmunology and can be adapted on a yearly basis.

At Amsterdam University College (AUC), the Netherlands, the need for such an undergraduate course was recognized a few years ago. Amsterdam University College is a broad liberal art and science honors college that provides a biomedical track. Since the College does not have laboratory facilities, these were provided by the local dentistry faculty, the Academic Center for Dentistry Amsterdam (ACTA), University of Amsterdam, and Vrije Universiteit (VU) Amsterdam together with the Cellular Imaging microscopy facility of the Amsterdam Medical Center (AMC), University of Amsterdam. We took up the challenge to design a flexible course that can be adapted per year, thus meeting our own desire to be able to line-up with current research of the department. This article tells the story of such a course set-up in the emerging field of osteoimmunology, but in essence, its structure can be applied, and adapted to any immunology course. The course we describe here, Cell Biology, and Physiology Lab, is an existing course, but can be adapted on a yearly basis. The course is evaluated every year at AUC, allowing for improving it further. We have experience in adapting it per year, some of its results can be used by PhD students, or senior scientists from the department.

For scientist-teachers, who are obliged to dedicate some of their precious time to the supervision of a practical course, this time is often considered as “lost,” especially when it concerns the supervision of a practical course that is repeated year-after-year without adapting it. Time not spend on own research is lost time, that is a common perception at university. To motivate scientist-teachers that there was some scientific gain in it as well, our course was designed in such a way that the theme of the course connected to the own research interest of the scientist-teachers. Some of the results obtained by students who were for the first time in a laboratory environment could be used, if supervised properly, in research papers of the scientist-teachers. For students, this stirs up the exciting realization that they are involved in cutting-edge research. “You will be the ones who, for the first time will discover ….” Therefore, the benefit for the two stakeholders, students and scientist-teachers, of our tailor-made, and yearly adapted course is symbiotic.

First of all, for students, the course will teach them how to put together solid research data for the different figures of a so-called “almost ready manuscript” at the end of the course. It gives them an appreciation of how to view a central process in immunology research from the perspective of the outcomes of 6 to 7 techniques. They will learn to integrate findings obtained by these techniques. At the end of such a course, students know how to generate, analyze, and weigh results. On top of that, they have gained appreciation of both the excitement of new results and the blood-sweat-and-tears that is inevitably involved in scientific research. Secondly, for the other group of stakeholders, the scientist-teachers, time spent on the course becomes useful time since it contributes not only to leaving a lasting impression on the students, but also to progressing the field and the research progress of the department.

## The Scientific Background for the Course: Osteoimmunology and Experimental Periodontology

Around the year 2000, it became more and more clear that the immune system and bone cells communicate. Inflammatory cytokines were shown to activate osteoclasts; T-cells were documented as either contributing to bone loss or to temper bone loss. It was discovered that T-cells and osteoclast precursor cells share transcription factors. Bacterial products were shown to influence both osteoblasts being the bone builders, and osteoclasts being the bone degraders. And after all, osteoclasts were then already known for 20 years to be derived from hematopoietic cells, more precisely from monocytes. This has led to the coining of the term “Osteoimmunology” (1–3) and also, recently, to redefining cells like osteoclasts as not only degraders of bone, but also as immune cells (4–6).

Periodontitis, the chronic inflammatory disease with loss of the tooth-surrounding bone, is the most common inflammatory bone disease. It is estimated that ~46% of American adults of 30 years and older have periodontitis, 3.8% of the Americans have a severe form of periodontitis (7). Its etiology comprises the presence of periodontopathogenic bacteria, such as *Porphyromonas gingivalis*, that interact with the cells from the tooth-surrounding tissue, the periodontium, and evoke an inflammatory reaction. Within the tissue, cells will recognize bacterial components with so-called pattern recognition receptors, of which the Toll-like receptors (TLRs) are widely studied (8). In particular, TLR2, and TLR4 are important in recognizing the periodontopathogenic component (9, 10). The recognition of bacterial components evokes an inflammatory response, causing the release of inflammatory cytokines such as interleukin-1β, and TNF-α, attracting a diversity of immune cells to the periodontium. This influx of leukocytes was characterized both in mice (11), reviewed in de Vries et al. (12), and in humans (13, 14). The sequential influx may consist of various innate immune cells such as neutrophils, and monocytes, and at a later stage T-cells from subsequently the Th1, Th2, Th17, and Treg classes, and finally plasma cells that make antibodies against components of the periodontopathogenic bacterial components that may invade the tissue. When enduring, these bacterial components present in the periodontium will eventually activate the monocyte-derived bone degrading cell, the osteoclast. This cell will then degrade the rims of bone between teeth, ultimately leading to tooth loss.

These cellular and bacterial interactions can be mimicked in an immunology/cell biology laboratory. Cells from the periodontium, especially fibroblasts, can be retrieved from extracted wisdom teeth. This surgical waste material is very valuable for the type of research described here. A rim of cells can be retrieved at the occlusal side. This is called the gingiva. More apically, the periodontal ligament can be scraped off the tooth root, and periodontal ligament fibroblasts can be grown from these tiny tissue fragments. The periodontal ligament anchors teeth into bone. The gingiva is the tissue closer to the tooth-epithelium connection and plays a role in anchoring epithelium to the bone, epithelium to tooth by collagenous fibers. The fibroblasts from these tissues, together with peripheral blood mononuclear cells, can be used for the differentiation of osteoclasts (15). Fibroblasts are considered to provide the cytokines macrophage colony stimulating factor (M-CSF) and the osteoclast differentiation factor receptor activator of NF-kappa ligand (RANKL) (16). Gingiva and periodontal ligament fibroblast cultures can be infected with *Porphyromonas gingivalis* to study the induced expression of inflammatory cytokines (17). Recently, it was shown that gingiva fibroblasts not only provide stimuli for osteoclast formation They also retain leukocytes and contribute to the T-cell proliferation as assessed by carboxyfluorescein succinimidyl ester (CFSE) labeling (18).

Apart from their role in catabolic processes, such as osteoclast formation, tooth-associated fibroblasts may also play a role in the regeneration of degraded bone (19). When cultured with vitamin C, needed for proper collagen folding, and with β-glycerophosphate as phosphate source, mineralization nodules are formed (20, 21). Therefore, gingiva or periodontal ligament fibroblasts represent an attractive model to study the effect of external influences on both anabolic and catabolic processes within the same experiment (22, 23).

Noteworthy, these fibroblasts may perceive chronic bacterial stimuli at a periodontitis-affected site of a tooth. Therefore, it is desirable to develop models that mimic such a chronic burden and assess both anabolic or osteogenic on the one hand and catabolic or osteoclastogenic effects on the other hand. Ideally, a co-culture with for instance the periodontitis-associated biofilm could be used, but chronic exposure to bacteria will kill cells in assays that last 21 days and this exposure is likely not biologically relevant, since such high encounter of bacteria likely does not take place within tissues. Biologically more relevant as a chronic exposure model, is the use of defined bacterial cell wall fragments that may leak into the tissue and that specifically target for instance TLR2 or TLR4, so-called TLR agonists. By using specific compounds instead of whole bacteria, it can be determined which activated TLR causes what effect.

## The Osteoimmunology Experiment Mimicking a Chronic Infection

The experiments for the course were prepared ~1 month in advance. Gingival fibroblasts from six donors were retrieved from a liquid nitrogen tissue collection of cells cultured from non-inflamed extracted wisdom teeth. These were propagated for ~1 week until a 175 cm^2^ tissue culture flask was confluent at the beginning of the experiment. One day before the start of the experiment, fibroblasts were seeded for osteogenesis or osteoclastogenesis experiments in 48 wells plates. The next day, day 0 of the experiment, either osteogenic medium, or peripheral blood mononuclear cells isolated from a buffy coat were added as previously described in detail (22–24). For the experiments assessing the effects of TLR activation on osteoclast activity, CD14+ monocytes were isolated using MACS technology (25), and seeded on top of bone slices. Experimental conditions were TLR2 agonist (PAM2, a synthetic diacylated lipopeptide; Invivogen, San Diego, CA) or TLR4 agonist (ultrapure LPS from *Porphyromonas gingivalis*; Invivogen, San Diego, CA) or a combination of both, previously titrated (Gerasimos Karlis, TJdV). In total, the experiment lasted 21 days. Cell cultures were refreshed twice a week with culture medium containing solvent or TLR2 or TLR4 or TLR2+4 agonists and supernatant for ELISA was taken every week. Samples were taken throughout the experiment, either by fixing the cells (for confocal, SEM, osteoclast microscopy, or Alizarine red staining), or by lysing the cells by RNAlysis buffer (qPCR), or water (alkaline phosphatase and DNA) or a lysis buffer for biochemical assays. Samples were stored at 4, −20, or −80°C. To assess the effect of TLR agonists on cell proliferation of peripheral blood mononuclear cells, PBMCs were labeled before experiments with CFSE as described previously (18). Proliferation assays making use of CSFE labeling were analyzed once a week at days 7, 14, and 21. Briefly, cells were detached with trypsin/EDTA and stained with the appropriate cell markers to enable linkage of CSFE fluorescence to leukocyte origin.

## The Course of Course: Integrating 7 Techniques in a Coherent Way

**Figure 1:**
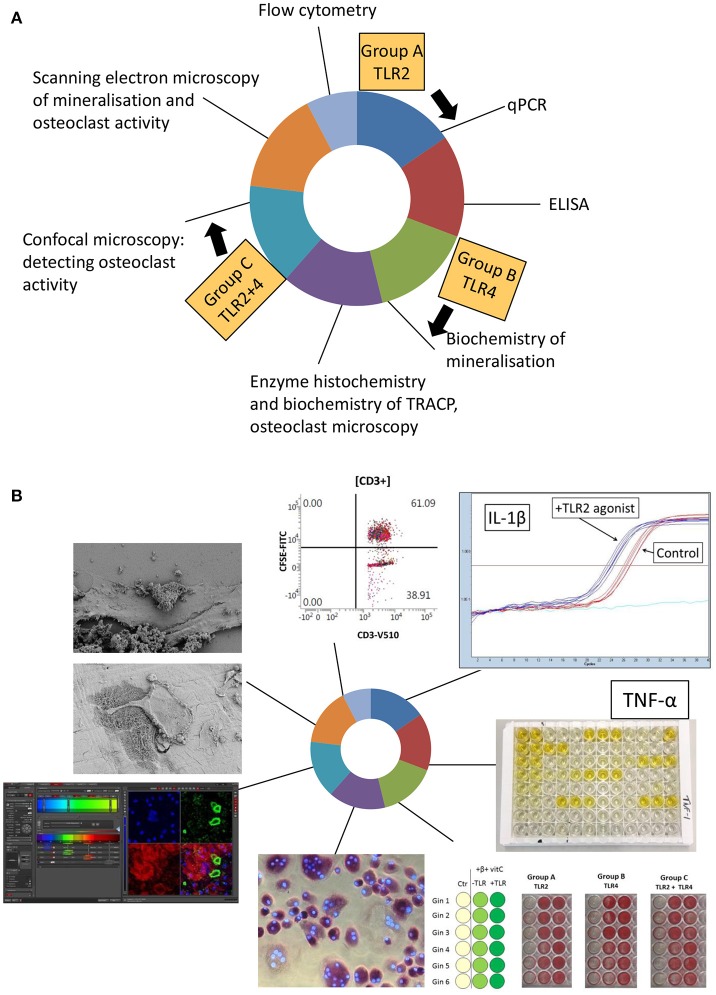
Set-up of the osteoimmunology course. **(A)** Over time, 6 techniques were visited by the three groups, group A (TLR2 agonist), group B (TLR4 agonist), and group C (TLR2+TLR4 agonist) for 2 days in a row. Flow cytometry was demonstrated during a 1 day master class. In principle, the order of these techniques is not relevant, provided that instructors take the time at the beginning of each technique introduction to emphasize the links with the previously examined techniques. **(B)** Illustrated outcomes of the course. All micrographs and graphics were taken during the course.

**Figure 2:**
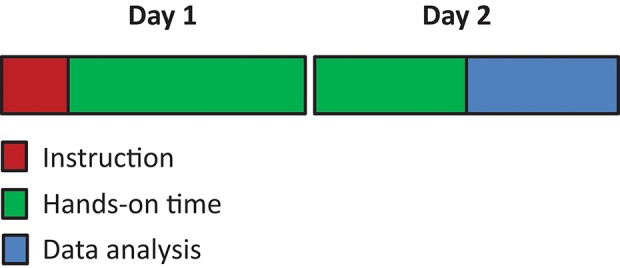
The typical sequence for a 2 day technical training. All 2 day techniques modules started with short instruction of the technique followed by ~1 day of hands-on training. The last few hours of day 2 were used for data analysis and interpretation.

Students enrolled for the course a few month before. A few weeks before the start of the course, students were informed on the theme and the learning objectives of the course. At an introductory morning session, students, and instructors were first introduced to one another where after three groups were formed. After a brief introduction on the theme of the course, groups were assigned to analyze the different experimental conditions. Group A would analyze all aspects of TLR2, group B of TLR4, and group C of the combination of TLR2, and TLR4. Each group also analyzed control samples. For the next 3 weeks, groups attended 6 technique modules (Figure 1). Each module lasted 2 days and was supervised for these 2 days by the corresponding technique supervisor. To ensure that consistent and usable results were obtained, the same technique supervisor supervised all three groups. Per technique, a short introduction into the technique and instruction for the following 2 days by the instructor preceded the hands-on laboratory work, and the data analysis (Figure 2).

### Research Questions per Technique Module

The course and the research questions were set-up in such a way that the order of visits of the 6 techniques (Figure 1A) was not relevant for fitting the pieces of the scientific puzzle together in the last week of the course.

Research questions per module were formulated beforehand. These are summarized in Box 1.

BOX 1 **Techniques and research questions per technique.**

- **qPCR:**
  What is the effect of TLR activation on gene expression in osteoclast cultures?
- **ELISA:**
  What is the effect of TLR activation on the secretion of inflammatory cytokines?
- **TRACP enzyme quantification, TRACP staining and microscopy:**
  Does TLR activation influence osteoclast formation?
- **Alkaline phosphatase enzyme, Alizarin red staining and calcium deposition:**
  What is the effect of TLR activation on bone formation?
- **Confocal microscopy:**
  Does TLR activation influence osteoclast activity (1)?
- **Scanning electron microscopy:**
  Does TLR activation influence osteoclast activity (2)?
  Does TLR activation lead to differences in mineral deposits?
- **Flowcytometry workshop:**
  Does TLR activation influence T-cell proliferation?

### Keyword = Coherence: Per Technique, Between Techniques and Between Experimental Variables

When mimicking scientific research within the course, there should be scientific coherence between its different modules. No technique was carried out just for the sake of the technique. Findings per technique should be compared with outcomes obtained using other techniques, hereby refining, and testing outcomes from multiple perspectives. We thus sought to link the various techniques, encouraging students to find scientific relationships at the end of the course, after completing all modules. Coherence was thus deliberately incorporated in the course. Here, we list 5 examples of coherence (Box 2), either within a technique module, between the modules, and between experimental variables (TLR2, TLR4, and TLR2+4 agonists), representing the overall outcomes between groups A–C.

BOX 2 **Examples of coherence.**

**Example 1:**

The production/secretion of the pro-inflammatory cytokine IL-1β was measured by ELISA and by qPCR. Did both techniques give a similar result? What is the interpretation of possible differences?

**Example 2:**

The effect of TLR activation on mineralization was measured using three techniques (alkaline phosphatase, Alizarin Red staining, as well as calcium deposition over time). These techniques also connected to the analysis of mineral deposits using SEM. Students were invited to find coherence between the techniques, but also to note the effect of TLR activation on differentiation over time.

**Example 3:**

For the analysis of tartrate resistant acid phosphatase (TRACP)-positive multinucleated cells, both cell counts as well as TRACP enzyme analysis were combined; TLR activation here resulted in fewer osteoclasts and corresponded to less TRACP activity.

**Example 4:**

There are basically two ways to detect bone resorption using microscopy. Osteoclasts grown on bone slices can be fixed and subsequently bone resorption pits can be assessed using SEM. Alternatively, actin ring formation, typical of resorbing osteoclasts, can be researched using confocal microscopy. These resorption results of the two techniques should in principle be complementary: *in situ* activity can only be shown with actin rings using confocal microscopy, while SEM should be used to study resorption in conjunction to osteoclasts. Examples of the practical are shown in Figure 1B.

**Example 5:**

Having gathered all the data at the end of the third week, students were able to formulate overall effects of TLR2, or TLR4 or by the combination of both on osteoclast formation or on osteogenesis. Furthermore, by connecting per technique to the group members of the other groups, specific TLR2, or TLR 4 or TLR2+4 effects on for example TNF-α expression (ELISA) could be worked out.

After completing all techniques, time was reserved in the course schedule to meet with the supervisor for individual/personal assistance and feedback on data acquisition, analysis, and interpretation. Students were encouraged to do so, since we (as course supervisors) thought it essential that students can consult us at a very accessible way.

## Educational and Pedagogical Considerations

When designing the above-described course, we carefully thought about a variety of educational and pedagogical considerations. What are the features of the teacher-instructors for this course, how are they instructed? Should we evaluate the student's laboratory skills (e.g., pipetting skills)? How do we balance the theoretical and practical aspects of this course? How can we then evaluate the students for this course? How and when should we provide feedback?

### The Teachers-Instructors

In contrast to any purely theoretical course, teacher-instructors of this practical course should above all be experienced in laboratory work and should enjoy explaining the designated technique, even three times in a row. They should be skilled in interacting with a critical student audience. Above all this, and special for our course, since it was taught to a group of international students: all (Dutch) instructors should master the English language at a proficient level. Furthermore, all instructors were involved in grading the students (described later).

### Evaluate Pipetting Skills?

Students of the honors college AUC are used to a system of continuous assessment. This would mean ~3 to 4 assignments spread over the whole month. We decided to deviate from this format, since our immunology laboratory skills lab would benefit from putting all assignments at the end of the course, when all results would be available. Since all assignments we chose were at the end of the course, and since it is a laboratory course, it could be argued whether lab skills should be assessed. We contemplated grading pipetting skills (and some students suggested this) but finally decided against it; it did not seem just to evaluate manual dexterity as some of the students held a pipette for the first time in their life. Furthermore, the design of the course, with its 2 days per module format, did not allow for assessing independent mastery of performing a technique at the per-student-level. These aspects are typically assessed during laboratory internships, where usually several attempts with gradually declining supervision are required before independency is guaranteed.

### The Three Assignments

**Figure 3:**
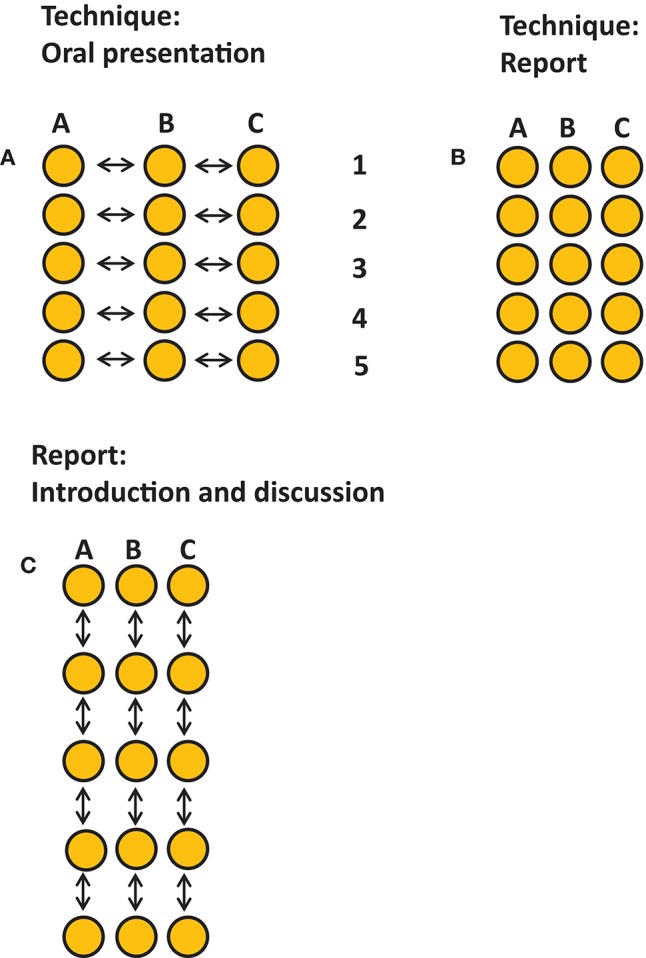
Graphical representation of the assignments. Three assignments took place, each accounted 1/3 of the final grade. Each dot represents one student, arrows indicate interactivity. **(A)** An oral presentation on the results per technique describing the ins-and-outs per technique, techniques 1–5, and the joint outcomes per technique per group presented by representatives for each group. This assessment promoted consultation and collaboration between group members of each group. **(B)** An individual assignment describing the ins-and-outs and the own results of one technique Five techniques were chosen to be covered in the report. **(C)** A group assignment for writing the introduction and discussion. This assignment promoted collaboration and interaction within the group.

There were three assignments (Figure 3). We decided to assess a presentation on the laboratory technique that was chosen by each student. Per laboratory technique, students of the three groups had to collaborate on this, comparing outcomes of TLR 2 (group A), TLR 4 (group B), or TLR 2+4 (group C) activation (Figure 3A). Each student had an individual assignment of writing up the results of the chosen technique (Figure 3B). Both for the oral evaluation and for the written assignment, students were instructed to introduce the purpose of the chosen technique, and to demonstrate that they master the principle of the technique. Students had to interpret the specific results of their group in the individual written report and had to interpret the results of the three groups during the oral presentation.

All individual reports were combined in the group report that should have the organization of a large research article on the effect of TLR 2 (group A), TLR4 (group B) or the activation of both (group C). As part of the final assignment, each group had to collaborate to write an introduction and a general discussion together. All three assignments counted for 1/3 of the final mark. Oral presentations were scored by all instructors, who were all invited to write down their feedback using a standardized feedback form containing the rubrics and the weight per rubric. Individually written technique was scored by the technique supervisor, and the group work introduction, and discussion were scored by two of the teachers. All three assignments had their own rubrics. The rubrics were known to the students in advance. Course instructors were instructed by the course coordinator (TJdV) on how to fill in the rubrics and how this would lead to the final mark per assignment.

### Performance Feedback to Students

Many summer courses, like ours, finish practically on the last days of the summer semester, leaving little room for feedback of the grading since students leave campus immediately after handing in their assignments. From a students' perspective, it is best to receive individual feedback. All remarks on group presentation were known directly after the presentation, but the written individual, and group assignments were marked in the week after the students had left. One person (TJdV) assembled all comments, and wrote a 2-page individualized report justifying the 3 grades that were obtained and sent those reports per email to each student.

## Student Course Evaluation

It is a good habit to evaluate all courses, both to the benefit of future students, and to the benefit of teachers. Therefore, student evaluations are a valuable instrument to assess quality and to initiate a plan-do-check-act (PDCA) cycle (26) to improve courses. In our view, course evaluations are only successful when filled in by a large group of participants. The questions of the evaluation were from a general format from the VU University Amsterdam, used by AUC. These included 13 questions on the course (i.e., learning outcomes, relevance, facilities, course information, learning outcomes achieved) 12 questions on the teachers (i.e., quality of teaching, command of English, variation of class activities, whether teacher encourage active participation), and 2 questions on the assessments (continuous assessment useful and whether assessments were a good reflection). Apart from these 1–5 scale evaluation questions, students were offered the possibility to reflect by typing their findings of the course in an open question format. To ensure unbiased and non-repetitive feedback, all students were encouraged to fill in the digital 1–5 scale evaluation form on their laptops prior to the informal evaluation in class. This way, a response rate of 87% (13 out of 15) was achieved.

Overall, the course scored higher than AUC average on 26 out of the 27 aspects that were evaluated. Six out of 27 aspects scored significantly higher than AUC average. Students especially appreciated the dedication and enthusiasm of the instructors, the variety of subjects and the meaningfulness in the broader perspective for the biomedical track. Awareness on how to organize and interpret research was raised. Among points of critique were the relatively late assessments (see The three assignments) and the too short introduction (half a day) of the theme of the laboratory course.

These are a few quotes of appreciation of the course. On a very positive note: “The instructors were super excited and motivated, which was amazing! They were very inspirational and motivated and provided very effective guidance in the lab.” Also: “I learned a lot and am happy about it.” And: “The atmosphere was great, and they obviously enjoy their work.” But then: “All instructors were good, however, the presentations before we started the practical work helped me understand what we were about to do. I would recommend that all instructors do the same in the future.” And, in the same line, more critical toward the variation between the teachers: “There were differences between teachers. I preferred when teachers explained the goal of the experiment in the beginning.” These points of critique, asking for a synchronized instruction of instructors, will further help improving the course. Also, more emphasis on the theoretical background of the experiment and repetition of this in the context of the technique, was already implemented this year. Finally, another valid point was on spending more time on data analysis: “When it comes to data analysis after the techniques, maybe it is already an idea to introduce graphpad to the groups at the beginning of the course so that little time can be spend on the same day as the experiment which saves time afterwards.” This point was taken up as well and the year following the course, more emphasis was placed on statistical analysis of data.

Instructors evaluated the course as well, but informally. This was done before the course started (feasibility of the specific assignment), during the course, and after the course. The level and enthusiasm of students was very much appreciated. Some teachers had a difficult time in bringing across the coherence between the techniques, which was picked-up a year later by putting more emphasis on this aspect. Finally, the course received peer-to-peer feedback by a course coordinator from another laboratory course at AUC.

## Conclusions

The course was to a great extent successful in bringing across new developments in the emerging field of osteoimmunology in a tangible way. Student stakeholders learned the essentials of commonly used immunology laboratory techniques in the context of a current hot topic in immunology with 6 modules of 2 days and a workshop on flow cytometry. Scientist-teacher stakeholders have benefitted from the course since some of the scientific outcomes (quantitative results from osteoclast counts, ELISA, mineralization assays, qPCR, and flow cytometry and illustrative SEM results) of the course will be used in two publications (G. Karlis et al., manuscript in preparation; C. Moonen et al., manuscript under revision). Quite a few students benefited from this course first of all by motivating them to apply for a laboratory internship, which was true for at least 4 out of 15 students in their third year. And, in general, years long experience has shown that experience obtained during laboratory courses such as this one, genuinely helps when applying for MSc programs and for applications to Medical Schools. Of equal importance: both stakeholders, students and teachers, have had an enjoyable, and even memorable time, therefore time well-spent!

## Data Availability

Requests to access the course evaluation data should be directed to the corresponding author at teun.devries@acta.nl.

## Author Contributions

Course coordinator TdV initiated writing. TS, HvV, JH, PK, CM, and IJ contributed textually and contributed to Figure 1B. All authors were instructors of the described course.

### Conflict of Interest Statement

The authors declare that the research was conducted in the absence of any commercial or financial relationships that could be construed as a potential conflict of interest.
